# Quorum Sensing Inhibition and Metabolic Intervention of 4-Hydroxycinnamic Acid Against *Agrobacterium tumefaciens*

**DOI:** 10.3389/fmicb.2022.830632

**Published:** 2022-03-07

**Authors:** Jin-Wei Zhou, Peng-Cheng Ji, Huan Jiang, Xiao-Juan Tan, Ai-Qun Jia

**Affiliations:** ^1^School of Food and Biological Engineering, Xuzhou University of Technology, Xuzhou, China; ^2^State Key Laboratory of Marine Resource Utilization in South China Sea, School of Life and Pharmaceutical Sciences, Hainan University, Haikou, China; ^3^School of Environmental and Biological Engineering, Nanjing University of Science and Technology, Nanjing, China; ^4^Anhui Provincial Key Laboratory of Molecular Enzymology and Mechanism of Major Diseases, Anhui Normal University, Wuhu, China

**Keywords:** *Agrobacterium tumefaciens*, quorum sensing, 4-hydroxycinnamic acid, metabolism, pathogenicity

## Abstract

The natural product 4-hydroxycinnamic acid (HA) was firstly isolated from the metabolites of *Phomopsis liquidambari*, one endophytic fungus from *Punica granatum* leaves. The anti-QS potential of HA was evaluated by β-galactosidase assay and acylated homoserine lactones (AHL) analysis. The MIC of HA was > 1.20 mM. Exposure to HA at sub-MIC concentrations (0.30–0.60 mM) remarkably reduced the β-galactosidase activity and AHL secretion. Transcriptional analysis by qRT-PCR and docking simulation indicated that HA functions as an anti-QS agent by inhibiting the transcriptional levels of *traI* and *traR* rather than signal mimicry. The blocked QS lead to suppressed biofilm formation, motilities, and flagella formation after exposure to HA at concentrations ranging from 0.30 to 0.80 mM. The dysfunctional QS also resulted in repressed antioxidant enzymes and intensified oxidative stress. The intensified oxidative stress destroyed membrane integrity, induced energy supply deficiency, resulted in disorder of protein and nuclear acid metabolism, and ultimately weakened pathogenicity of *A. tumefaciens*. HA may have promising potential for controlling *A. tumefaciens*.

## Introduction

*Agrobacterium tumefaciens* is a soil-borne phytopathogen and characterized by its ability to cause crown gall disease, a plant tumor affecting a wide range of plants (Lee et al., 2009). *A. tumefaciens* has the potential to infect more than 1,000 various species of dicotyledonous plants as well as some species of monocots, and many of them are agronomically important crops (Wang et al., 2018). Crown gall disease is responsible for extensive economic losses to nursery productions of fruits plants, roses, grapevines in many countries (Pulawska, 2010). *A. tumefaciens* infects plants by incorporation of its transfer-DNA (T-DNA) into genome of plant cells, and eventually results in the uncontrolled formation of tumors on account of the unbalanced auxin and cytokinin and uncontrolled cell division. The transfer of T-DNA is activated once *A. tumefaciens* detects phenols secreted by the wounded plant cells. Studies have shown that the horizontal transfer of T-DNA is tightly mediated by *vir* genes (Lang et al., 2013). Therefore, suppressing *vir* gene expression is a suitable approach for blocking T-DNA transfer, and would ultimately attenuate the tumorigenesis efficiency of *A. tumefaciens*.

It has been evidenced that the *vir* gene expression are encoded by the conjugative tumor-inducing (Ti) plasmid, and the dissemination of which was mediated by the quorum sensing (QS) signal 3-oxo-octanoyl-L-homoserine lactone (3-oxo-C8-HSL) (Lang and Faure, 2016). The QS signal 3-oxo-C8-HSL is synthesized by the TraI protein, a LuxI homologue encoded in Ti plasmid. 3-oxo-C8-HSL binds to the receptor TraR and the formed complex binds to the promoter elements *tra* boxes, eventually initiates T-DNA transfer and motivates virulence-related gene expression (Swiderska et al., 2001). Therefore, disrupting QS could be an attractive approach for controlling crown gall disease caused by *A. tumefaciens*.

Two basic approaches were employed for disrupting QS of *A. tumefaciens*, i.e., enzyme degradation and small molecules competitive binding (Uroz et al., 2009). The latter has been widely studied by applying chemically synthesized analog of the QS signals to bind to the receptors (Galloway et al., 2011). Though these synthetic analog have potential for acting as QS inhibitors, production costs and success rates make drugs from natural products more suitable (Kong et al., 2009). Endophytes are ubiquitous in all plant species and have proven to be potential sources of novel natural products and anti-QS agents for exploitation in medicine and agriculture (Yu et al., 2010). For example, a series of novel polyhydroxyanthraquinones with ant-QS potential were isolated from the metabolites of endophytic fungus *Penicillium restrictum* (Figueroa et al., 2014). The endophytic fungus *Alternaria alternata* produced bis (2-methylpropyl) ester, 2-propyl tridecyl ester and 1,2-benzenedicarboxylic acid, which possessed anti-virulence and anti-biofilm capacities (Rashmi et al., 2018). Recently, 4-hydroxycinnamic acid (HA) was firstly isolated from the secondary metabolites of *Phomopsis liquidambari* S47, one endophytic fungus obtained from the leaves of *Punica granatum*. The structure of HA was elucidated by ESI-MS and NMR. HA has anti-QS potential against *A. tumefaciens* (Bodini et al., 2009). However, the underlying anti-QS mechanism and metabolic response of HA to *A. tumefaciens* have not been pinpointed. In this study, the anti-QS potential of HA was assessed by employing the AHL-responsive reporter strain *A. tumefaciens* A136 (pCF218/PCF372) (*traI*: *lacZ*) (McLean et al., 1997) and the possible metabolic mechanism of HA was investigated through ^1^H NMR spectroscopy.

## Materials and Methods

### Isolation of the Metabolites With Quorum Sensing Inhibitory Activity

*P. liquidambari* S47 was preserved in China Center for Type Culture Collection (M2018476). After 15-d incubation in Fungus No. 2 medium (Zhou et al., 2017), the metabolites were extracted with ethyl acetate and then purified by normal and reversed phase silica gel chromatography, HPLC, and Sephadex LH-20 gel permeation chromatography with *A. tumefaciens* KYC55 (pJZ372, pJZ 384, pJZ410) as the activity tracing strain (Zhou et al., 2017). The purified metabolites were identified by ESI-MS and NMR.

### Growth Measurement

HA used in this study was purchased from Shanghai Yuanye Bio-Technology Co., Ltd. This compound is white needle crystal with purity > 98%. The minimum inhibitory concentration (MIC) of HA was determined using the broth microdilution method with an inoculum of 1–5 × 10^5^ CFU/mL (Molitor, 2019). The growth analysis was determined by inoculating 0.1% overnight cultures of *A. tumefaciens* into AT medium (Kim et al., 2005) supplemented with different concentrations of HA. After incubation at 28°C for 24 h, growth was determined by reading OD_620_. Each treatment was performed in triplicate.

### β-Galactosidase Activity

Overnight cultures of *A. tumefaciens* A136 (100 μL) were inoculated into 100 mL of AT medium with or without HA. The signal molecule 3-oxo-C8-HSL (3 μM) was added into the culture and then cultivated at 28°C for 17 h. Salicylic acid (SA, 0.10 mM) was used as the positive control. Each treatment was performed in triplicate. After cultivation, growth was evaluated by reading OD_620_ (A_620_). For β-galactosidase activity, 200 μL of cultures were added with 800 μL of Z-buffer, 100 μL of 0.1% SDS, 150 μL of chloroform, and 100 μL of ONPG (4 mg/mL). The mixture was incubated at 28°C for several minutes (T), followed by the addition of 1 M sodium carbonate solution. After centrifugation, the supernatant was determined by reading OD_420_ (A_420_). The β-galactosidase activity was evaluated using the following formula (Stachel et al., 1985).


(1) $$\frac{A_{420}\times 1,000}{A_{620}\times T\times 0.2\,m\,L}$$

### Biofilm Formation

Biofilm formation was assayed in 96-well plate as described by Merritt et al. (2005). Briefly, *A. tumefaciens* C58 was cultivated in AT medium with 0.30, 0.60, and 0.80 mM of HA at 28^°^C for 24 h. DMSO served as the negative control. After cultivation, the bacteria solution was removed and the planktonic bacteria were washed with PBS gently. Biofilms were dried at 60^°^C, fixed with 200 μL of methanol, stained with 100 μL of 0.05% crystal violet, washed with PBS to remove excess dye, and quantified after solubilization of the dye with ethanol by reading the microplates at 570 nm. For microscopic analysis, the biofilms established in 24-well chambered cover slides were washed with PBS, fixed with 2.5% glutaraldehyde, dehydrated with graded ethanol, and subsequently freeze-dried (Zhou et al., 2018b). Biofilms were then gold-coated, and subjected to scanning electron microscopy (SEM, JSM6360, JEOL, Tokyo, Japan).

### Acylated Homoserine Lactones Analysis

The anti-QS activity of HA was further confirmed by quantifying the 3-oxo-C8-HSL level secreted by *A. tumefaciens* C58 (wild strain) (Wood et al., 2001). Briefly, 0.1% overnight cultures of *A. tumefaciens* C58 was inoculated into AT medium with or without HA and then cultivated at 28^°^C for 17 h. Each treatment was performed in triplicate. After centrifugation, the supernatant was extracted with an equal volume of acidified ethyl acetate. After evaporation, the residues were dissolved with methanol and 3-oxo-C8-HSL was quantified using liquid chromatography-tandem mass spectrometry (LC-MS/MS) (SHIMADZU) equipped with a C18-column (250 × 4.6 mm, 5 μm, Shimadzu, Tokyo, Japan) (Zhou et al., 2018b). Mobile phase A was formic acid (0.1%) and ammonium formate (5.0 mM) in water while mobile B was MeOH. The flow rate was 0.4 mL/min with 10 μL injection volume. The gradient was 1–5 min, 20–50% B; 5–20 min, 50–90% B. The eluates were determined with a ThermoFinnigan LCQ Classic system (San Jose, CA) using the positive mode. The nebulizer was 15 psi, drying gas 7 mL/min, and temperature 300^°^C. Full-scan MS was from m/z 100 to 1,000. 3-oxo-C8-HSL was identified according to the fragment MS ions and the retention time of commercial standard (Sigma-Aldrich, United States). The area of the ion m/z 102 was selected to quantify 3-oxo-C8-HSL due to its specificity and better signal-to-noise ratio. The extracted ion chromatograms were employed for area calculation.

### Swimming and Chemotaxis

For swimming motility assay, 2 μL overnight cultures of *A. tumefaciens* C58 (OD_620_ = 0.5) were dropped to the ATGN medium with different concentrations of HA as described by Merritt et al. (2007). Salicylic acid (0.10 mM) and DMSO served as the positive and negative control, respectively. The swimming diameter was determined after 24-h cultivation at 28^°^C. Each treatment was performed in triplicate.

Chemotaxis assay was performed by inoculating 2 μL overnight cultures of *A. tumefaciens* C58 on one side of the ATGN medium (without glucose) with different concentrations of HA as described by Ding and Christie (2003). One strip filter paper dipped with glucose was set as 4 cm aside from the inoculated bacterial cultures. After 24-h incubation at 28^°^C, the chemotaxis diameter was measured. Each treatment was performed in triplicate.

### Flagella Formation

After 17-h cultivation with or without HA, 50 μL of 37% methanol was added to 1 mL of bacterial cultures. The mixture was centrifuged at 5,000 rpm for 2 min and washed with distilled water. The collected cells were resuspended with distilled water and then dropped on a slide. After 5-min staining with the flagella staining solution (Beijing Solarbio Science & Technology Co., Ltd., China), flagella were detected with a light microscope (Nikon 80i, Japan). Each treatment was performed in triplicate.

### Metabolic Analysis

*A. tumefaciens* C58 was cultivated with 0.60 mM of HA at 28^°^C for 17 h. After cultivation, cells were collected and washed three times with precooled PBS. Cell pellets were homogenized with 3.8 mL of methanol/H_2_O (1/0.9) and then added with 4 mL of chloroform. The mixtures were centrifuged and the upper layer was polled. After removal of methanol, samples were lyophilized and then resolved in D_2_O phosphate buffer for ^1^H NMR analysis. Data processing and metabolites assignment were performed as described previously (Zhou et al., 2019a). The fold change values of metabolites between groups were calculated by the ratios of integral non-overlapping areas of each metabolite. The Benjamini and Hochberg (BH) method was used to adjust the *p*-values for controlling the false discovery rate in multiple comparisons using scripts written in R language. The fold change values of metabolites and associated *p*-values adjusted by BH methods in different group comparisons were visualized in fold change plots. Each treatment was performed 11 times.

### Reactive Oxygen Species and H_2_O_2_ Measurement

For ROS determination, the harvested cells were mixed with 6-carboxy-2′,7′-dichlorodihydrofluorescein diacetate (DCFH-DA, 1 mM). After 30-min cultivation at 28^°^C, cells were collected, washed with distilled water, and then resuspended with PBS (1 mL). ROS was quantified using a Hitachi 2,700 fluorescence spectrophotometer (Hitachi, Japan) as described previously (Zhou et al., 2019b). Each treatment was performed in triplicate.

H_2_O_2_ was determined according to Gonzalez-Flecha and Demple with minor modifications (Gonzalez-Flecha and Demple, 1997). Briefly, bacterial cultures were pelleted and resuspended with PBS. Intracellular H_2_O_2_ passed through the membranes and equilibrated with the buffer. After 1-min centrifugation at 6,000 g, the suspensions were used for H_2_O_2_ analysis employing the horseradish peroxidase-scopoletin method. Each treatment was performed in triplicate.

### Quantitative Real-Time PCR

After cultivation, cells were harvested and total RNA was extracted using RNA extraction kit (Tiangen Biotech, Beijing, China). The Quantitative Real-Time PCR (qRT-PCR) assay was performed as described by Zhou et al. (2018b) with *lepA* (*atu0241*) set as the reference gene (Lang et al., 2016). The primers were listed in Supplementary Table 1. Each treatment was performed in triplicate.

### Docking Analysis

Discovery Studio 4.0 program was used for docking simulation. The structures of HA and 3-oxo-C8-HSL were performed by ChemBioDraw 12.0. The target TraR (NDB code PD0654) was docked with 3-oxo-C8-HSL and HA, respectively, and the scores we recorded as kcal/mol (Zhou et al., 2018a).

### Tobacco Infection Assay

Overnight cultures of *A. tumefaciens* C58 were inoculated into AT medium with 0.30 and 0.60 mM of HA. After cultivation at 28^°^C and 180 rpm for 17 h, cells were collected, washed with sterile distilled water, and then resuspended with PBS to obtain an OD_620_ = 0.1. Stems of 4-week-old tobaccos were inoculated with bacterial suspensions using a needle as described by Dandurishvili et al. (2011) and Liu et al. (2012). The black control was inoculated with sterile PBS. Photograph and tumor weight were recorded after inoculation for 4 weeks. Each treatment was performed in triplicate.

### Statistical Analysis

All assays were performed at least three times, and data were expressed as means ± standard deviation (SD). Graphs were constructed using Origin 8.6 software (OriginLab, Northampton, MA, United States). Students’*t*-test was performed using SPSS 18.0 software (SPSS, Inc., Chicago, IL, United States) for comparing differences between groups. A *p* ≤ 0.05, 0.01, and 0.001 were all considered statistically significant and indicated by *, **, and ***, respectively.

## Results

### Purification and Identification of Active Metabolites

A total of 61 compounds were purified from the metabolites of *P. liquidambari* by repeated silica gel, RP-18 gel, Sephadex LH-20, and HPLC with *A. tumefaciens* KYC55 as the reporter strain. Limited by their content and spectroscopic techniques, 10 compounds were elucidated by ESI-MS and NMR spectra. Among these 10 compounds, 4-hydroxycinnamic acid (HA) showed the strongest anti-QS capacity against *A. tumefaciens*. The spectroscopic data of HA were presented as follows.

4-hydroxycinnamic acid (Supplementary Figure 1): C_9_H_8_O_3_; ESI-MS *m/z* 163 [M-H]^–^; ^1^H NMR (600 MHz, DMSO-*d*_6_): δ_*H*_ 6.29 (1H, d, *J* = 16.0 Hz, H-8), 6.79 (1H, d, *J* = 8.5 Hz, H-3,5), 7.49 (1H, d, *J* = 16.0 Hz, H-7), 7.50 (1H, d, *J* = 8.5 Hz, H-2,6); ^13^C NMR (150 MHz, DMSO-*d*_6_): δ_*C*_ 115.3 (C-8), 115.8 (C-3,5), 125.3 (C-1), 130.1 (C-2,6), 144.2 (C-7), 159.6 (C-4), 168.0 (C-9)(Qianbo et al., 2010).

### Minimum Inhibitory Concentration Determination

**FIGURE 1:**
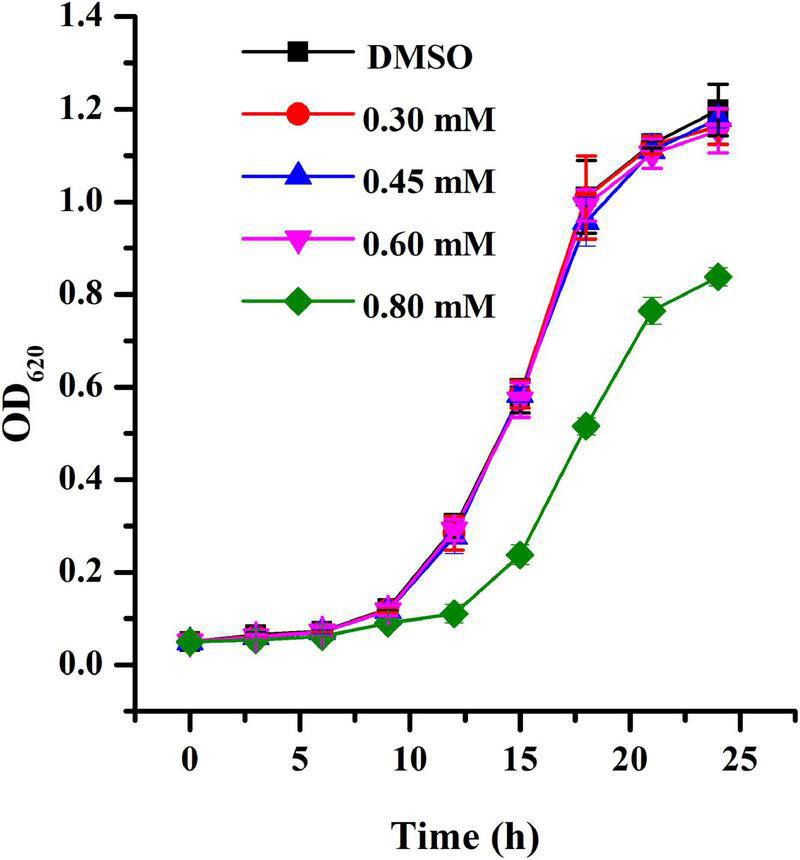
Growth profile of *A. tumefaciens* treated with HA. Growth was evaluated at various concentrations of HA (0.30, 0.45, 0.60, and 0.80 mM) for 24 h. DMSO was used as the negative control. Error bars represent standard deviations of three measurements.

The MIC of HA against *A. tumefaciens* was > 1.20 mM. The growth curve was obtained using HA at sub-MIC concentrations. Results indicated that exposure to HA at 0.30, 0.45, and 0.60 mM had no inhibitory impact on cell growth (Figure 1). However, treatment with HA at 0.80 mM showed inhibitory effect on cell growth (Figure 1).

### β-Galactosidase Activity

**FIGURE 2:**
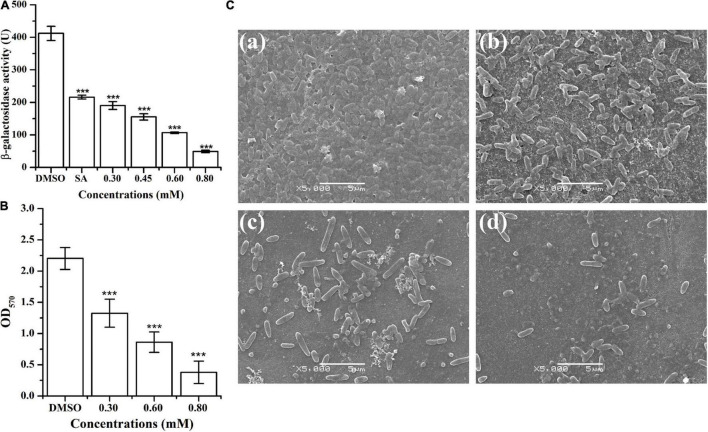
Effect of HA on β-galactosidase activity and biofilm formation of *A. tumefaciens*. **(A)** Effect of HA (0.30, 0.45, 0.60, and 0.80 mM) on β-galactosidase activity using *A. tumefaciens* A136 as the AHL-responsive reporter strain. Salicylic acid (SA, 0.10 mM) and DMSO were used as positive and negative control, respectively. **(B)** Effect of HA (0.30, 0.60, and 0.80 mM) on biofilm formation. **(C)** SEM images of *A. tumefaciens* treated with **(a)** DMSO, **(b)** 0.30 mM, **(c)** 0.60 mM, and **(d)** 0.80 mM of HA. Statistical differences were determined by Student’s *t*-test. ***p < 0.001 vs. the DMSO control.

The ani-QS activity of HA was determined through β-galactosidase activity using *A. tumefaciens* A136 (*traI*::*lacZ*) as the AHL-responsive reporter strain. Results indicated that treatment with HA at 0.30, 0.45, 0.60, and 0.80 mM reduced β-galactosidase activity by about 54, 62, 74, and 88%, respectively (Figure 2A). The anti-QS activity of HA was more effective than that of salicylic acid, whose exposure resulted in 48% inhibition of β-galactosidase activity.

### Biofilm Formation

The inhibitory effect of HA on *A. tumefaciens* biofilm formation was presented in Figures 2B,C. Treatment with HA at concentrations of 0.30, 0.60, and 0.80 mM significantly reduced the formation of biofilms by 40, 61, and 83%, respectively (Figure 2B). In addition to quantitative analysis, treated biofilms were also visualized using SEM. The SEM images showed thick biofilms in the control experiment (Figure 2Ca), whereas HA exposure at 0.30, 0.60, and 0.80 mM significantly removed the microbes attached to the glass surface (Figures 2Cb–d).

### Acylated Homoserine Lactones Production

**FIGURE 3:**
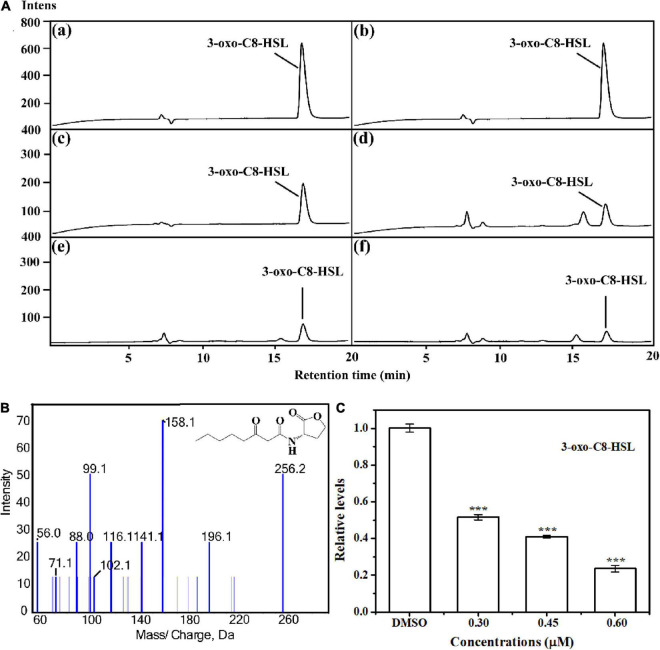
Relative quantification of 3-oxo-C8-HSL by LC-MS/MS chromatograms. **(A)** HPLC chromatograms of 3-oxo-C8-HSL produced by *A. tumefaciens* C58 treated with **(c)** DMSO and **(d–f)** HA (0.30, 0.45, and 0.60 mM, respectively). **(a)** and **(b)** represented the standard chemicals of 3-oxo-C8-HSL. **(B)** MS/MS spectra of 3-oxo-C8-HSL. **(C)** Quantitative analysis of 3-oxo-C8-HSL treated with 0.30, 0.45, and 0.60 mM of HA, respectively. Statistical differences were determined by Student’s *t*-test. ***p < 0.001 vs. the DMSO control.

The anti-QS activity was further confirmed by determining AHL level produced by *A. tumefaciens*. Results indicated that the signal molecule 3-o-C8-HSL was secreted in the supernatant of *A. tumefaciens* C58 (Figures 3A,B). Treatment with HA at 0.30, 0.45, and 0.60 mM remarkably reduced the peaks and areas of 3-oxo-C8-HSL (Figure 3A). Relative quantification by HPLC chromatograms indicated that HA treatment resulted in the reduced production of 3-oxo-C8-HSL by about 50, 60, and 80%, respectively (Figure 3C).

### Motilities and Flagella Formation

**FIGURE 4:**
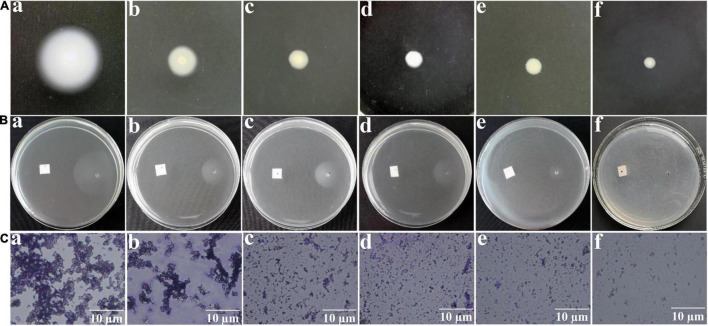
Effect of HA on swimming motility **(A)**, chemotaxis **(B)**, and flagella formation **(C)**. Images of **(a–f)** represented DMSO, salicylic acid (SA, 0.10 mM), 0.30, 0.45, 0.60, and 0.80 mM of HA treatment, respectively. The images of swimming motility and chemotaxis were obtained by a common camera. The flagella formation was observed using a light microscope.

Swimming, chemotaxis, and flagella are essential for *A. tumefaciens* to survive for nutrients and infect host (Erhardt, 2016). Therefore, interfering with swimming, chemotaxis, and flagella formation may suppress the infection ability and reduce the pathogenicity of *A. tumefaciens*. Results indicated that exposure to HA at 0.30, 0.45, 0.60, and 0.80 mM, the swimming motility (Figure 4A), chemotaxis (Figure 4B), and flagella formation (Figure 4C) of *A. tumefaciens* were remarkably suppressed. The suppressed ability of HA was superior to that of salicylic acid.

### Metabolic Profile

**FIGURE 5:**
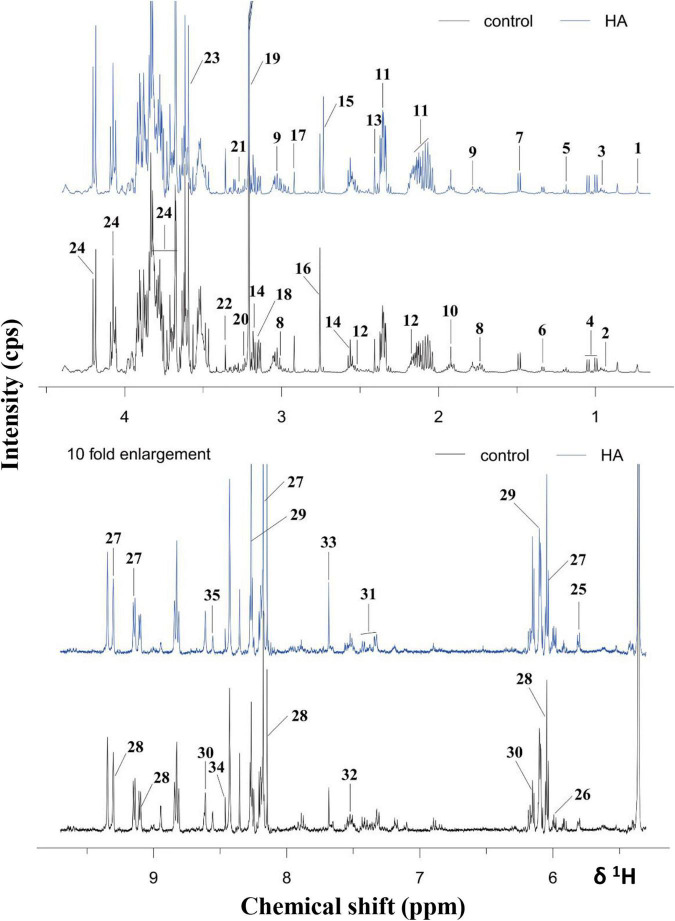
^1^H NMR spectra of *A. tumefaciens* extracts from AHE-treated (red line) and control group (black line). Labeled metabolites: cholate (1), isoleucine (2), leucine (3), valine (4), ethanol (5), lactate (6), alanine (7), lysine (8), putrescine (9), acetate (10), glutamate (11), glutathione (12), succinate (13), β-alanine (14), dimethylamine (15), sarcosine (16), *N*, *N*- dimethylglycine (17), ethanolamine (18), choline (19), arginine (20), betaine (21), methanol (22), glycine (23), sucrose (24), uracil (25), UDP-galactose (26), NAD^+^ (27), NADP^+^ (28), adenosine (29), AMP (30), phenylalanine (31), tryptophan (32), xanthine (33), formate (34), and ATP (35).

**FIGURE 6:**
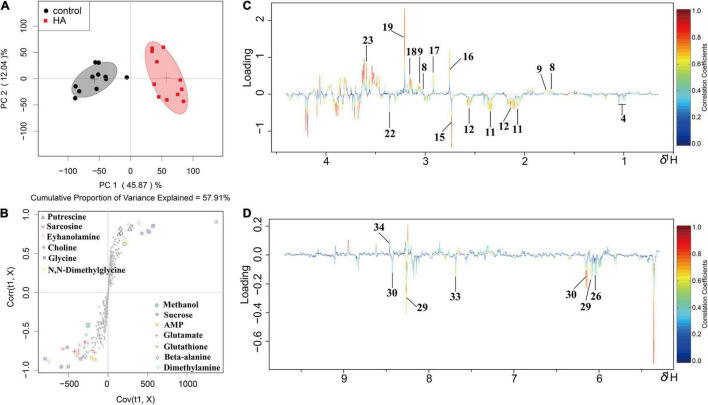
OSC-PLS-DA of metabolomics profiles from HA-treated and control groups. **(A)** PCA score plot. **(B)** S-plot points represent different variables (metabolites). **(C,D)** Color-coded loading plot after removal of water signals and affected regions. The color bar was applied in which red and blue represented metabolites that statistically significantly or indistinctively contributed to the separation of groups, respectively. Peaks in positive and negative status revealed decreased and increased metabolites relative to score plot in the HA-treated group.

^1^H NMR-based metabolomics approach was employed to evaluate the metabolic changes of *A. tumefaciens* C58 exposure to HA (Figure 5). A remarkable decrease in cholate, putrescine, sarcosine, *N*, *N*-dimethylglycine, ethanolamine, choline, glycine, and formate, and a significant increase in glutamate, glutathione, dimethylamine, methanol, UDP-galactose, adenosine, AMP, and xanthine were observed after HA treatment (Table 1 and Figures 6B–D). The PCA score plot presented a significant separation between HA and the DMSO-treated groups (Figure 6A). This indicated that the metabolism of *A. tumefaciens* C58 was deeply disturbed after HA treatment. The detailed assignment of metabolites and their fold changes were displayed in Table 1.

**TABLE 1:** Important metabolites assignments in *A. tumefaciens* C58, their fold change values, and associated *p*-values.

No.	Metabolites	Assignments	Chemical shift^a^ (ppm)	Fold change^b^	*P*^c^ value
1	Cholate	CH_3_	0.74 (s)	−0.12	*
2	Isoleucine	δ-CH_3_, β-CH_3_	0.94 (t)	0.04	
3	Leucine	δ-CH_3_, CH_2_	0.97 (t)	0.02	
4	Valine	CH_3_, CH_3_	1.0 (d),1.045 (d)	0.32	*
5	Ethanol	CH_2_	1.18 (t)	0.26	*
6	Lactate	CH_3_	1.34 (d)	0.23	
7	Alanine	CH_3_	1.485 (d)	0.00	
8	Lysine	δ-CH_2_, α-CH	1.71 (m), 3.02 (m)	−0.18	*
9	Putrescine	2N-CH_3_	1.79 (m),3.05 (m)	−0.42	***
10	Acetate	CH_3_	1.92 (s)	−0.16	
11	Glutamate	β-CH_2_, α-CH_2_, N-CH	2.07 (m),2.35 (m)	0.44	***
12	Glutathione	CH_2_	2.18 (m),2.52 (m)	0.47	*
13	Succinate	CH	2.41 (s)	0.19	*
14	β-Alanine	CH_2_	2.58 (t),3.18 (t)	−0.05	
15	Dimethylamine ine	CH_3_	2.74 (s)	3.65	***
16	Sarcosine	CH_3_	2.755 (s)	−0.82	***
17	*N*, *N*- Dimethylglycine hylglycine	N-CH_3_	2.92 (s)	−0.78	***
18	Ethanolamine ne	N-CH_2_, CH_2_	3.15 (t)	−0.88	***
19	Choline	N(CH_3_)_3_	3.21 (s)	−0.76	***
20	Arginine	CH_3_	3.24 (t)	−0.09	
21	Betaine	α-CH_2_	3.27 (s)	−0.22	*
22	Methanol	CH_3_	3.36 (s)	1.07	**
23	Glycine	α-CH_2_	3.595 (s)	−0.25	***
24	Sucrose	Furan ring CH	3.67 (m),3.84 (m),4.08 (t)	0.07	
25	Uracil	Pyridine ring CH	5.8 (d),7.5 (d)	−0.08	
26	UDP-galactose ose	Furan ring CH	6.00 (d)	0.66	***
27	NAD^+^	Pyridine ring CH	8.19 (s),9.14 (d),9.34 (s)	−0.03	
28	NADP^+^	Pyridine ring CH	8.14 (d),9.095 (s),9.305 (s)	0.13	
29	Adenosine	Furan ring C_3_H	6.08 (d),8.35 (s)	0.30	*
30	AMP	2′-CH	6.14 (d),8.61 (s)	0.46	***
31	Phenylalanine ne	H_2_/H_6_	7.38 (m)	0.01	
32	Tryptophan	C_7_H, C_4_H	7.55 (d)	0.28	
33	Xanthine	CH_3_	7.68 (d)	0.53	*
34	Formate	CH	8.46 (s)	−0.47	*
35	ATP	2′-CH	8.555 (s)	−0.13	

*^a^Multiplicity: (s) singlet, (d) doublet, (t) triplet, (m) multiplet.*

*^b^Color-coded according to the log_2_(fold): red and blue represent the increased and decreased metabolites, respectively, in HA-treated group.*

*^c^P-values were calculated based on a parametric Student t-test or a nonparametric Mann-Whitney test and were corrected by the BH (Benjamini-Hochberg) methods.*

Values with asterisk symbols denoted extent of significance: *p < 0.05, **p < 0.01, and ***p < 0.001.

### Reactive Oxygen Species and H_2_O_2_ Analysis

**FIGURE 7:**
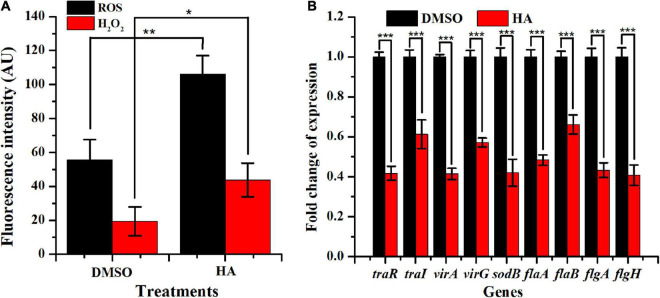
Effect of HA at 0.60 mM on oxidative stress **(A)** and expressions of genes involved in QS, virulence, antioxidant enzyme, and flagella formation **(B)**. Student’s *t*-test was used to calculate *p*-values (two-tailed). *p < 0.05 vs. the DMSO control; **p < 0.01 vs. the DMSO control; ***p < 0.001 vs. the DMSO control.

The production of ROS and H_2_O_2_ with HA treatment was displayed in Figure 7A. HA exposure at 0.60 mM remarkably enhanced the production of ROS and H_2_O_2_. The result implied that *A. tumefaciens* C58 underwent severe oxidative stress after exposure to HA.

### Gene Expressions

qRT-PCR was used to evaluate the impact of HA (0.60 mM) on the transcriptional levels of nine genes *traR*, *traI*, *virA*, *virG*, *sodB*, *flaA*, *flaB*, *flgA*, and *flgH*, which were involved in QS, virulence, antioxidase, and flagella formation. Results indicated that HA treatment lead to a remarkable suppression on the expressions of *traR* (∼59%), *traI* (∼39%), *virA* (∼59%), *virG* (∼43%), *sodB* (∼58%), *flaA* (∼51%), *flab* (∼34%), *flgA* (∼56%), and *flgH* (∼59%) (Figure 7B). The obtained data indicated that HA treatment resulted in the breakdown of QS, lead to enhanced oxidative stress, and would inevitably reduce the pathogenicity of *A. tumefaciens* C58.

### Docking Simulation

**FIGURE 8:**
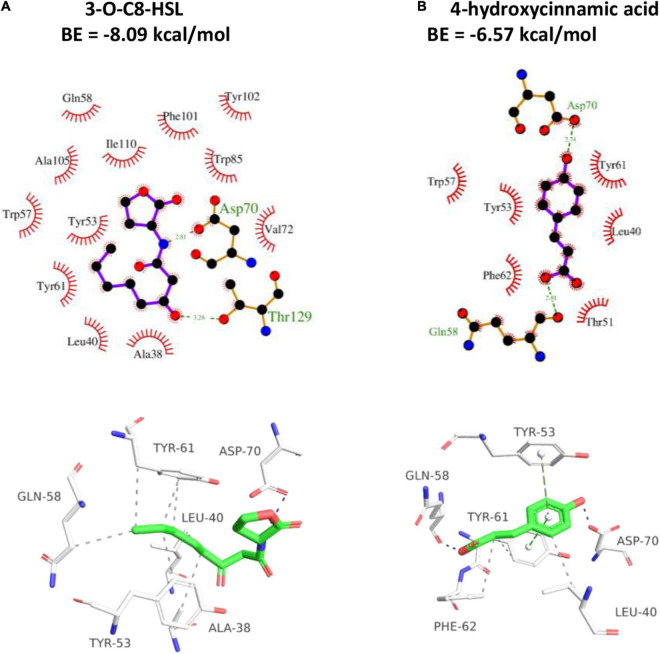
2D (top) and 3D (bottom) schematics of receptor-ligand interactions of TraR with 3-oxo-C8-HSL **(A)** and HA **(B)**, respectively, using the Discovery Studio 4.0 program.

Docking simulation was employed for assessing the proposed binding domain and binding ability of HA to TraR. The amino group of 3-oxo-C8-HSL showed H-binding interaction with Asp70 at a distance of 2.81 Å and the carbonyl group exhibited H-binding interaction with Thr129 at a distance of 3.26 Å (Figure 8). The two hydroxyl groups of HA displayed H-interactions with Asp70 and Gln58, respectively (Figure 8). The binding energy between 3-oxo-C8-HSL and TraR was −8.09 kcal/mol, whereas HA was −6.57 kcal/mol. The result indicated that 3-oxo-C8-HSL has stronger binding affinity to TraR than HA.

### Pathogenicity Suppression Assay in Tobacco

**FIGURE 9:**
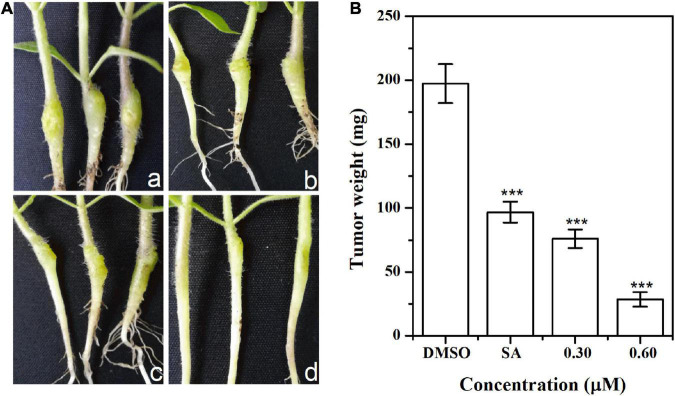
Effect of HA on tobacco plant infections. **(A)** Inoculation with *A. tumefaciens* C58 treated with **(a)** DMSO, **(b)** salicylic acid (SA, 0.10 mM), **(c)** 0.30 mM of HA, and **(d)** 0.60 mM of HA, respectively. **(B)** Quantification of crown gall weight treated with or without HA. Statistical differences were determined by Student’s *t*-test. The F values of SA and HA (0.30 and 0.60 mM) analysis were 1.000, 1.318, and 0.028, respectively. The df values of all treatments were 4. ***p < 0.001 vs. DMSO-treated control.

The inhibitory effect of HA on pathogenicity was evaluated by inoculating *A. tumefaciens* C58 into tobacco stems. Results indicated that the size and weight of crown galls from infection with HA-treated *A. tumefaciens* C58 were evidently smaller compared with the untreated control (Figure 9). The tumorigenic ability of the HA-treated strain was obviously attenuated. The results indicated that HA had the potential to function as a pesticide in treating *A. tumefaciens* infection.

## Discussion

*A. tumefaciens* is a soil-borne phytopathogen that infects a wide range of dicotyledonous plants by transferring and integrating its own T-DNA into plant genome (Gohlke and Deeken, 2014). The expressions of the integrated genes intensified the synthesis of auxin and cytokinin, and then induced the proliferation of crown galls, activated the expressions of virulence-related genes, and ultimately caused the reduction of crop yield. It has been evidenced that T-DNA transfer and virulence activation were well correlated with QS (Fuqua and Winans, 1994). Thus, suppressing QS would be a compelling alternative for controlling crown gall disease caused by *A. tumefaciens*. In this study, a QS inhibitor named 4-hydroxycinnamic acid (HA) was firstly isolated from the metabolites of *P. liquidambari* S47 and its anti-QS, anti-virulence, and metabolic mechanisms were determined.

The potential anti-QS activity of HA was firstly evaluated by β-galactosidase assay. The reduced β-galactosidase activity indicated that HA might disrupt the QS of *A. tumefaciens*. As the significance of AHL in coordinating conjugal transfer of the tumor-inducing plasmid and mediating the expressions of a battery of genes involved in virulence (Swiderska et al., 2001), the effect of HA on AHL secretion was measured. The notable suppression of AHL secretion further indicated the potent anti-QS activity of HA. The result was consistent with the reduced transcriptional levels of *traI* and *traR*. However, docking analysis indicated that the binding affinity of HA to TraR was weaker than that of 3-oxo-C8-HSL. Therefore, we speculated that the anti-QS potential of HA was obtained by inhibiting *traI* and *traR* expressions rather than competitive binding.

In addition, biofilms, flagella and motilities are essential for drug resistance, sensing plant-derived signals, striving for nutrients, and infection process (Ding and Christie, 2003). Our result showed that HA treatment resulted in a significant inhibition in biofilm formation, swimming, chemotaxis, and flagella formation. The inhibition of biofilm formation would reduce the resistance of pesticides. The suppressed motility and flagella formation would inevitably result in reduced virulence and infection efficiency of *A. tumefaciens*. These results were further confirmed by qRT-PCR analysis and tobacco infection assay, which showed suppressed transcriptional levels of *virA*, *virG*, *flaA*, *flaB*, *flgA*, and *flgH*, and reduced crown gall disease after exposure to HA.

The impact of HA on metabolic profile of *A. tumefaciens* was deeply evaluated by ^1^H NMR-based analysis. Results presented that the metabolic system of *A. tumefaciens* was significantly disturbed with HA exposure. Some of the altered metabolites were related to quorum sensing, protein synthesis, and oxidative stress, including putrescine, valine, ethanolamine, and choline. It has been evidenced that the synthesis of putrescine was deeply affected by QS signals (Zhu et al., 2016). The reduced putrescine was consistent with the inhibited β-galactosidase activity and reduced AHL secretion. Valine is one of the branched-chain amino acids and acts as vital roles in maintaining normal functions of cells (Zhou et al., 2019a). The changed valine might indicate the changed cell function caused by HA. Ethanolamine is an essential component of membrane and act as an important role in maintaining membrane integrity (Zhou et al., 2019a). The notable reduction in ethanolamine indicated the intensified oxidative stress caused by HA. To defend against oxidative damage and repair the damaged membrane, the consumption of ethanolamine was enhanced. This speculation was validated by the increased ROS and H_2_O_2_ levels. In addition, the significant reduction of choline, *N*, *N*-dimethylglycine, and sarcosine, and the increase of dimethylamine, glutamate, and glutathione also confirmed the intensified oxidative damage.

It is known that excessive oxidative damage can lead to cell death. However, in this study, HA treatment at 0.60 mM had no inhibitory impact on cell growth, which was evidenced by the growth profile, plate counting assay (Supplementary Figure 2A) and ATP investigation test (Supplementary Figure 2D). The orange cells (Supplementary Figure 2B) and enhanced EB fluorescence (Supplementary Figure 2C) suggested two possibilities: (1) cell death, or (2) the compromised integrity of cell membrane. Since HA is not bactericidal at 0.60 mM and the fluorescence intensity in HA-treated group was just 18% higher than that of DMSO-treated group, we can draw a conclusion that HA treatment destroyed the integrity of cell membrane. In addition, ROS was determined after treatment with the lethal concentration of 1.20 mM (data not shown). HA exposure at 1.20 mM resulted in the ROS production of 501 ± 37 fluorescence intensity, which was significantly higher than that of 0.60 mM of HA treatment (106 ± 11 fluorescence intensity). Thus, we speculated that the bactericidal effect of HA on *A. tumefaciens* is due to the excessive oxidative damage, while HA at sub-lethal concentration brought no excessive oxidative damage. In other words, the oxidative damage caused by HA at 0.60 mM was not enough to kill cells, but only changed the membrane composition and enhanced cell membrane permeability without sterilization. This speculation was in accordance with the study of Zhou et al. (2018b), who reported that the quorum sensing inhibitor hordenine could notably enhance the susceptibility of the preformed biofilms to ciprofloxacin by changing the permeability of membranes, as evidenced by the reduced level of ethanolamine. In addition, Cheng et al. (2020) also reported that the quorum sensing inhibitor dimethylaminocinnamic acid could destroy the integrity of cell membrane by reducing ethanolamine level with no sterilization.

Choline is the component of phospholipids and is responsible for keeping integrity and permeability of membrane (Zhou et al., 2019a). The reduced choline level indicated that the oxidative stress caused by HA destroyed the structure of membrane. To maintain oxidative balance and repair the damaged membrane, choline consumption was enhanced. The decomposition of choline resulted in the enhanced level of dimethylamine as dimethylamine was the downstream metabolite of choline (Dumas et al., 2006). The enhanced oxidative stress was further evaluated by determining the levels of glutamate and glutathione, and the transcriptional level of gene *sodB*. It has been proven that the activity of superoxide dismutase (SOD) was mediated by QS (Hassett et al., 1999). In this study, the transcriptional level of *sodB* encoding SOD was significantly repressed and this result was in line with the dysfunctional QS. The enhanced glutamate and glutathione and the suppressed *sodB* would inevitably intensified oxidative stress and ultimately lead to the damage of cell membrane, disorder of metabolic profile, and reduced pathogenicity of *A. tumefaciens*.

In addition, it has been evidenced that 3-oxo-C8-HSL synthesis is affected by fatty acid metabolism (Dong et al., 2017). The intermediate products of fatty acid metabolism can provide the matrix for 3-oxo-C8-HSL synthesis. The suppressed secretion of 3-oxo-C8-HSL indicated that fatty acid metabolism might be inhibited. The inhibition of fatty acid metabolism would change cell membrane composition and this was verified by the reduced ethanolamine and choline.

As a powerful antioxidant in organism, betaine acts as an important role in maintaining cell integrity and normal function (Chen et al., 2017). Betaine can be transformed into *N*, *N*-dimethylglycine with the catalysis of betaine homocysteine methyltransferase, and *N*, *N*-dimethylglycine was then transformed into sarcosine and glycine. The decrease of betaine can be attributed to choline reduction as betaine is the oxidative product of choline in organisms. To defend against oxidative stress and repair the damaged cells caused by free radicals, betaine was excessively consumed.

HA treatment also caused disorder of energy metabolism, which was evidenced by the increased succinate. Succinate is an essential intermediate involved in tricarboxylic acid (TCA) cycle. The altered succinate would inevitably block TCA cycle. As the most important pathway for producing energy, the block of TCA cycle would inevitably affect energy metabolism, lead to energy deficit, result in dysfunction of cells, and ultimately reduce virulence and pathogenicity of *A. tumefaciens*. This hypothesis was confirmed by the attenuated crown gall disease. The blocked TCA cycle and insufficient energy supply lead to intensified anaerobic respiration for compensation, which was supported by the increased level of ethanol.

Adenosine, AMP and xanthine are intermediate metabolites of purine nucleotide. AMP can be converted to adenosine by dephosphorylation, and then transformed into inosine by deaminase. Inosine is subsequently transformed into xanthine through hydrolysis and oxidation (Zhou et al., 2019a). As is known that AMP is derived from cyclic AMP (cAMP) through cAMP-specific phosphodiesterase and cyclic 3′,5′-phosphodiesterase. It has been evidenced that cAMP contributes to QS, biofilm formation, motility, and expressions of genes responsible for flagella, toxins, and energy supply (Liang et al., 2007). The increase of inosine, AMP, and xanthine indicated that the catabolism of cAMP was intensified. The excessive catabolism of cAMP would lead to dysfunctional QS and reduced pathogenicity of *A. tumefaciens*. This speculation was supported by the reduced AHL, suppressed virulence factors, and weakened infection in plant.

**FIGURE 10:**
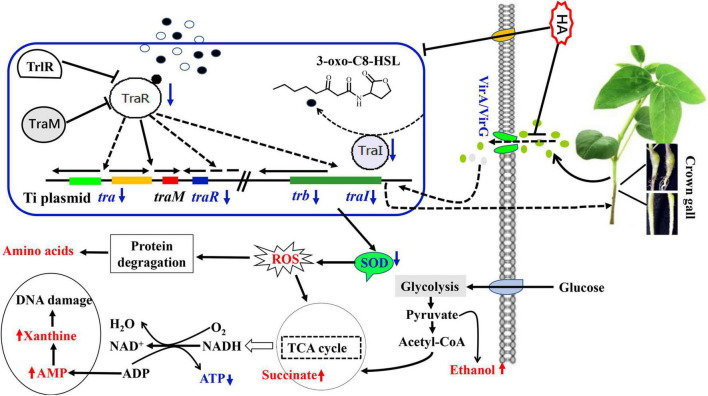
Schematic diagram of the metabolic pathways disturbed by HA. The red font indicated promoted pathways while blue font indicated inhibited pathways.

In sum, 4-hydroxycinnamic acid (HA) was firstly isolated from the metabolites of endophytic fungus *P. liquidambari* and its anti-QS, anti-virulence potential and metabolic mechanism were deeply investigated. A schematic diagram of the disordered metabolic mechanism was displayed in Figure 10. HA treatment resulted in the dysfunction of QS. The dysfunctional QS intensified oxidative stress, caused deficiency of energy supply, lead to disorder of protein metabolism and nuclear acid metabolism, and ultimately reduced virulence and crown gall disease caused by *A. tumefaciens*. Therefore, HA may act as an efficient pesticide for controlling *A. tumefaciens* infection.

## Data Availability Statement

The original contributions presented in the study are included in the article/Supplementary Material, further inquiries can be directed to the corresponding author/s.

## Author Contributions

J-WZ, A-QJ, and X-JT conceived, designed the experiments, and wrote the manuscript. J-WZ, P-CJ, and HJ performed the experiments and analyzed the data. All authors contributed to the article and approved the submitted version.

## Conflict of Interest

The authors declare that the research was conducted in the absence of any commercial or financial relationships that could be construed as a potential conflict of interest.

## Publisher’s Note

All claims expressed in this article are solely those of the authors and do not necessarily represent those of their affiliated organizations, or those of the publisher, the editors and the reviewers. Any product that may be evaluated in this article, or claim that may be made by its manufacturer, is not guaranteed or endorsed by the publisher.
