# Supplementary material for: Quorum Sensing Inhibition and Metabolic Intervention of 4-Hydroxycinnamic Acid Against Agrobacterium tumefaciens
Source: Front Microbiol. 2022 Mar 7;13:830632. doi: 10.3389/fmicb.2022.830632 (PMC8940537; doi:10.3389/fmicb.2022.830632)
Supplement: Supplementary file 1 [file Data_Sheet_1.docx]

**Journal:** Frontiers in Microbiology

Supplementary Material

**Quorum sensing inhibition and** **metabolic intervention of** **4-****hydroxycinnamic acid against *Agrobacterium tumefaciens***

Jin-Wei Zhou^1^, Peng-Cheng Ji^1^, Huan Jiang^2,3^, Xiao-Juan Tan^4,*^, Ai-Qun Jia^2,*^

^1^School of Food and Biological Engineering, Xuzhou University of Technology, Xuzhou 221018, China;

^2^School of Life and Pharmaceutical Sciences, State Key Laboratory of Marine Resource Utilization in South China Sea, Hainan University, Haikou 570228, China;

^3^School of Environmental and Biological Engineering, Nanjing University of Science and Technology, Nanjing 210094, China;

^4^Anhui Provincial Key Laboratory of Molecular Enzymology and Mechanism of Major Diseases, Anhui Normal University, Wuhu 241000, China.

*** Corresponding authors**

A.-Q.J: E-mail: [jiaaiqun@gmail.com](mailto:jiaaiqun@gmail.com). Tel: +86-898-66254967. Fax: +86-898-66254967.

X.-J.T: Email: xjtan@ahnu.edu.cn.


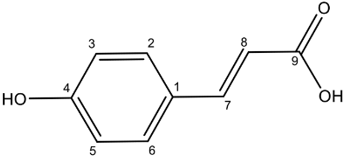


**Fig. S1.** Chemical structure of 4-hydroxycinnamic acid (HA).


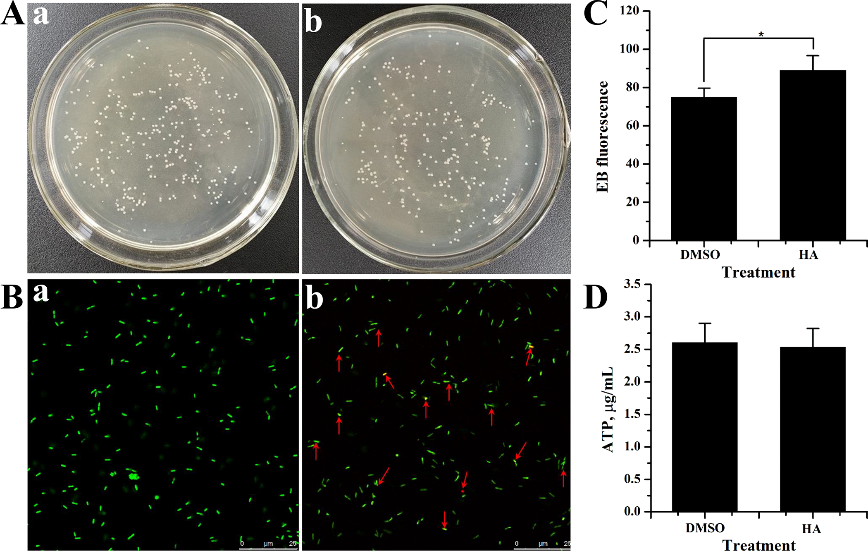


Figure S2. Effect of HA on growth measurement and membrane integrity. (A) Growth was measured by plate counting method treated with (a) DMSO and (b) 0.60 mM of HA. (B) CLSM images of AO/EB stained cells treated with (a) DMSO and (b) 0.60 mM of HA. (C) Quantification of EB fluorescence treated with DMSO and 0.60 mM of HA using Synergy H1 Hybrid Multi-Mode Microplate Reader. (D) Quantification of ATP concentrations in the cultures treated with DMSO and 0.60 mM of HA using the ATP assay kit (Nanjing Jiancheng Bioengineering Institute, China).

**Supplementary Table 1.** PCR primers for qRT-PCR.

| Genes | Primer direction | Sequence (5’-3’) | Amplicon size (bp) |
| --- | --- | --- | --- |
| *traR* | Forward | TATCCAGCACAGGCACATCA | 196 |
|  | Reverse | GGATGCGTGGTCATAGAAGG |  |
| *traI* | Forward | ATGGGATGTCGCAATAGAGG | 128 |
|  | Reverse | GTCCTGATGGCGCAAGAA |  |
| *virA* | Forward | ACCCTTCTTTACGACACGAGC | 163 |
|  | Reverse | TACGGGTTCCTTAGAAGACGG |  |
| *virG* | Forward | TAATCTCCTGGTTGCTTTCCTG | 201 |
|  | Reverse | TCAAAGAAATAGCCAGCACCT |  |
| *sodB* | Forward | TGGGAACACTCCTATTACATCG | 89 |
|  | Reverse | ACGTAGTCCCAGTTGATGAGGT |  |
| *flaA* | Forward | GCGGAAGAGCGACAGGATGTTC | 106 |
|  | Reverse | CGAAGAGTCCACCAAGCTGAAG |  |
| *flaB* | Forward | AGGATGCTTTCGGAGTTGCTGTTG | 114 |
|  | Reverse | CCTCGTGGACGCTGACATGAAC |  |
| *flgA* | Forward | ATGTCTGCTTGGAGATCATGCCTTC | 97 |
|  | Reverse | GAACAGGTGAAGTCGGTGGAAGTG |  |
| *flgH* | Forward | GTCAGGATGTCGCCGATATTGAGTG | 136 |
|  | Reverse | AATTCAGCCAGACACCGCAGATG |  |
| *lepA* | Forward | AGTATCCCGCTCGCTTTCA | 124 |
|  | Reverse | CGTGACCAGCTCGTGATTATT |  |
